# The Role of Bcl11 Transcription Factors in Neurodevelopmental Disorders

**DOI:** 10.3390/biology13020126

**Published:** 2024-02-17

**Authors:** Franziska Anna Seigfried, Stefan Britsch

**Affiliations:** Institute of Molecular and Cellular Anatomy, Ulm University, Albert-Einstein-Allee 11, 89081 Ulm, Germany; franziska.seigfried@uni-ulm.de

**Keywords:** Ctip1, Ctip2, Bcl11a, Bcl11b, brain development, neurodevelopment, neurodevelopmental disorders, disease modeling, transcription factor

## Abstract

**Simple Summary:**

Neurodevelopmental disorders are typically attributed to abnormal brain development. These disorders encompass a wide range of neuropsychiatric symptoms that can result in varying degrees of mental, physical, and economic consequences for the affected individuals, their families, and society. Most treatments available today are symptomatic. To develop more curative therapeutic approaches, it is necessary to identify the precise cellular and molecular mechanisms underlying neurodevelopmental disorders. Recently, mutations in *BCL11A* and *BCL11B*, two ultra-conserved zinc-finger transcription factors, have been associated with multiple cases of neurodevelopmental disorders, including developmental delay, autism spectrum disorder, intellectual disability, and structural brain alterations. Model organisms revealed Bcl11 transcription factors to be critical regulators of nervous system development. They are involved in neural progenitor cell proliferation, migration, differentiation, and synapse formation. Targeted mutations of *Bcl11a* and *Bcl11b* in mice result in phenotypic features remarkably similar to the corresponding disorders observed in humans. This makes animal models valuable tools for a better understanding of pathogenic mechanisms. This review provides a comprehensive overview of our current understanding of the functions of Bcl11 transcription factors in brain development. It links fundamental experimental research with the emerging amount of clinical and genetic data from individuals affected by Bcl11a- and Bcl11b-dependent neurodevelopmental disorders.

**Abstract:**

Neurodevelopmental disorders (NDDs) comprise a diverse group of diseases, including developmental delay, autism spectrum disorder (ASD), intellectual disability (ID), and attention-deficit/hyperactivity disorder (ADHD). NDDs are caused by aberrant brain development due to genetic and environmental factors. To establish specific and curative therapeutic approaches, it is indispensable to gain precise mechanistic insight into the cellular and molecular pathogenesis of NDDs. Mutations of *BCL11A* and *BCL11B*, two closely related, ultra-conserved zinc-finger transcription factors, were recently reported to be associated with NDDs, including developmental delay, ASD, and ID, as well as morphogenic defects such as cerebellar hypoplasia. In mice, Bcl11 transcription factors are well known to orchestrate various cellular processes during brain development, for example, neural progenitor cell proliferation, neuronal migration, and the differentiation as well as integration of neurons into functional circuits. Developmental defects observed in both, mice and humans display striking similarities, suggesting Bcl11 knockout mice provide excellent models for analyzing human disease. This review offers a comprehensive overview of the cellular and molecular functions of Bcl11a and b and links experimental research to the corresponding NDDs observed in humans. Moreover, it outlines trajectories for future translational research that may help to better understand the molecular basis of Bcl11-dependent NDDs as well as to conceive disease-specific therapeutic approaches.

## 1. Introduction—Background

### 1.1. Neurodevelopmental Disorders (NDDs)

The development of the human brain is a precisely orchestrated and timed process that continues after birth. It is influenced by genetic programs as well as the surrounding environment [1,2,3]. Substantial deviations from the typical trajectory may result in disturbed neuronal architecture or connectivity, leading to abnormal brain functions. NDDs are characterized by a disruption in the establishment of the central nervous system (CNS) due to imbalanced growth regulation processes [4].

NDDs encompass a range of symptoms that depend on neurodevelopment and coincide with impaired brain development that persist throughout an individual’s lifetime. The ‘International Statistical Classification of Diseases and Related Health Problems’ (ICD-11), which was approved by the World Health Organization in 2019, denotes the following disorders to the spectrum of NDDs: disorders of intellectual development; developmental speech or language disorders; ASD developmental learning disorder, including dyslexia and dyscalculia; developmental motor coordination disorder; attention deficit hyperactivity disorder (ADHD); and stereotyped movement disorder (Table 1) [5]. This diversity of symptoms and their severity have psychological, physical, and economic consequences for individuals, their families, and society [6,7]. Currently, most available therapies are limited to the unspecific treatment of symptoms of NDD. To develop more specific, curative therapeutic approaches, it is necessary to better understand the precise cellular and molecular mechanisms underlying the pathogenesis of NDDs.

To date, various factors have been identified as underlying causes of NDDs. These factors can be categorized as either intrinsic, such as genetic variations, or extrinsic, including environmental influences, toxification, and infection. One approach to identifying these key mediators is the investigation of potential genetic causes of NDDs. Some NDDs occur cryptogenically; however, several NDD-causative genes have been identified [4,8]. The SysNDD database annotated over 1600 high-confidence NDD genes, and those genes are active during early brain development and in several biological pathways, such as nervous system/synaptic function, transport, and chromosome/chromatin organization [8,9]. Often, gene families are linked to specific NDDs; for example, genetic variants in the solute carrier (SLC) group of membrane transport proteins (i.e., SLC6A1, SLC22A5, SLC25A13, SLC38A11) are associated to ASD in multiple cases [10]. The members of the calcium-dependent adhesion protein class of cadherins (CDH) that are involved in cellular contacts and migration throughout the development of the nervous system (i.e., CDH1-4, CDH7-8) are associated with NDDs, too [10]. More molecular pathways are affected in NDDs, including genes of the mTOR pathway in protein synthesis or genes of cell-adhesion molecules (CAMs), such as *NEUREXINs* and *SHANKs* in the organization of pre- and postsynaptic compartments [4]. Furthermore, the chromatin remodeling complex BRG1/BRM-associated factor (BAF) complex plays a critical role in the development of NDDs. A group of NDDs with high locus and phenotypic heterogeneity resembles the so-called BAFopathies, with mutations in genes encoding for BAF subunits [4,11]. Among these subunits, BCL11A and BCL11B, the members of the BCL11 transcription factor (TF) family, exert crucial roles in regulating neurodevelopmental processes [12,13,14].

This review provides an overview of current knowledge on connections between Bcl11 TFs and NDDs and gives an outline of future directions for building translational bridges to improve our knowledge of molecular regulations underlying NDDs.

### 1.2. Bcl11 Transcription Factor Family

The Bcl11 (B-cell leukemia/lymphoma 11) TF family consists of Bcl11a and Bcl11b, two extremely similar Krüppel-like C_2_H_2_ zinc-finger protein TFs [15,16]. Bcl11 proteins are ultra-conserved in evolution, with 95% homology between humans and mice [17]. The Bcl11 TFs share six homologous zinc-finger domains that are conserved from fruit flies to humans [18,19]. Due to genome duplication in vertebrate phylogeny, *Danio rerio* harbors duplicates of Bcl11a and Bcl11b. They are named Bcl11aa and Bcl11ab, as well as Bcl11ba and Bcl11bb. Bcl11 TFs are predominantly found in the lymphohematopoietic and central nervous system but also play important roles in skin integrity, tooth development, and suture closure [17,20,21,22,23,24]. Bcl11a and Bcl11b were originally described within the immune system, where they interact with chicken ovalbumin upstream promoter (Coup) TF orphan nuclear receptors and, therefore, are also known as Ctip1 and Ctip2 (CoupTF-interacting proteins 1 and 2, respectively) [16]. A profile of the Bcl11 TFs is given in Table 2, listing further synonyms and substantial details of the two TFs. Bcl11 TFs directly and/or indirectly interact with DNA to repress or enhance the expression of target genes [25]. Furthermore, Bcl11 TFs can build homo- and heterodimers using an N-terminal C_2_HC zinc-finger domain. This is of functional relevance for the translocation of different Bcl11 isoforms from the cytosol toward the nucleus [26,27]. In humans, there exist at least four relevant isoforms of BCL11A and two of BCL11B, respectively. BCL11A-XL and BCL11A-L, the two long BCL11A isoforms, feature two zinc-finger domains important for DNA interaction and localization to the nucleus. The short isoforms (BCL11A-S, BCL11A-XS) lack DNA-binding zinc-finger domains and are predominant in the cytoplasm. BCL11A-S can build heterodimers with long isoforms and, thereby, be transferred to the nucleus [28,29]. In the CNS, Bcl11 TFs are predominantly expressed in the hippocampus, striatum, and dorsal spinal cord, as well as in the olfactory, cerebral, entorhinal, and cerebellar cortex [20]. Further information on the existing isoforms, their molecular structure, and known binding complexes is described in detail by Simon et al. [30].

The locus of *BCL11A* is one of the most constrained regions within the human genome [31]. This is highlighted by the probability of being loss of function intolerant (pLI) reported in the gnomAD database for both *BCL11A* and *BCL11B*. pLI is calculated by an algorithm that takes the reported prevalence of gene variations, meaning the actual occurrence of a certain gene variation, and the mathematically determined prevalence of gene variations into account. A pLI approaching one (pLI ≥ 0.9) indicates an extremely inflexible set of transcripts [32]. The pLI values of *BCL11A* as well as *BCL11B* were predicted by gnomAD as 0.97 and 0.99, respectively, implying the gene loci to be extremely intolerant towards genetic changes [32]. The significance of Bcl11a and Bcl11b is furthermore demonstrated by the fact that knockout in mice leads to perinatal and postnatal lethality, respectively [33,34]. In Bcl11a knockout mice, the establishment of a functional immune system is disrupted, as B cells and several types of T cells fail to develop [33]. Moreover, Bcl11b is also critical for the specification and differentiation of alphabeta T cells, as the absence of Bcl11b results in an arrest of immature T cells in their CD4-; CD8-double negative state [34,35].

In the hematopoietic system, Bcl11a represses fetal hemoglobin (HbF) expression, thereby shifting the transition from HbF to adult hemoglobin (HbA) in maturing erythroid cells [36]. Therefore, BCL11A is a potential therapeutic target for reactivation of HbF in beta-hemoglobin disorders, including sickle cell disease and beta-thalassemia [36]. To find therapeutic concepts for the downregulation of BCL11A, several approaches, including CRISPR-Cas9 (clustered, regulatory interspaced, short palindromic repeat/CRISPR-associated protein 9) editing as well as the application of zinc finger nucleases or lentiviral vectors were investigated [37,38,39]. An erythroid enhancer element of *BCL11A* was defined that makes cell type-specific modification of *BCL11A* expression feasible [40,41]. Just recently, this enhancer region was targeted in the very first approved CRISPR-Cas9-related treatment in the United Kingdom [42,43].

To date, whole exome sequencing (WES) offers a powerful approach to exploring the genetic etiology of NDDs. Trio WES is performed on parent–child triplets to identify de novo mutations. Using WES, variants of *BCL11A* and *BCL11B* were reported to cause NDDs in several cases. Patients exhibit NDD-associated disorders of varying severity, such as developmental delay, ASD, and ID, in concert with morphological changes in the brain [13,14,44]. Connections between Bcl11 TFs—their role in brain development—and the manifestation of NDDs will be reviewed in the following chapters.

## 2. Role of Bcl11 TFs in CNS Development and NDDs

### 2.1. BCL11A-Related NDDs

In humans, the *BCL11A* gene locus is associated within the Online Mendelian Inheritance in Man (OMIM) database with two diseases, namely ‘Intellectual developmental disorder with persistence of fetal hemoglobin’ (OMIM: 617101) and ‘Chromosome 2p16.1-p15 deletion Syndrome’ (OMIM: 612513). The former is also known as ‘Dias-Logan Syndrome’ as well as ‘BCL11A-related Intellectual Disability’ (BCL11A-IDD) and displays genetic changes exclusively in the *BCL11A* gene. The latter represents larger deletions in the gene regions encompassing *BCL11A.* Clinical features of these diseases embrace a spectrum of neurological origin, such as ID, speech impairment, epilepsy, and behavioral issues [13,45].

In general, individuals with the chromosome 2p16.1-p15 deletion syndrome develop more severe phenotypes than patients with BCL11A-IDD [46]. Miceli et al. summarized the situation in a total of 38 patients (36 previously published and 2 newly investigated) [47]. Brain magnetic resonance imaging (MRI) of individual patients shows increased structural alternations, such as dysplasia, hypoplasia, and atrophy in neural structures, including the corpus callosum, cortex, cerebellum, pontine, and optic nerve [45,46,47,48]. Moreover, patients feature phenotypes, including hearing loss, intrauterine growth restriction, and short stature, that cannot be delineated to variations in the *BCL11A* gene but are associated with *USP34* (ubiquitin specific peptidase 34) and *XPO1* (exportin 1), two candidate genes in close proximity [47].

In 2014, a patient with a microdeletion restricted to the *BCL11A* gene was described with neurodevelopmental delay, severe speech disorder, and attention deficit, thereby delineating these traits for the first time specifically to *BCL11A* [49]. Recently, Peron et al. provided a comprehensive collection of clinical features as well as genotype–phenotype correlations in BCL11A-IDD [50,51]. They performed an in-depth analysis of 75 patients (42 newly studied patients and 33 previously described patients). In this cohort, 60 unique variants of *BCL11A* were compared, including 30 frameshift, 7 missense, 6 splice-site, and 17 stop-gain variants, as well as 8 unique microdeletions in *BCL11A*. 

**Figure 1:**
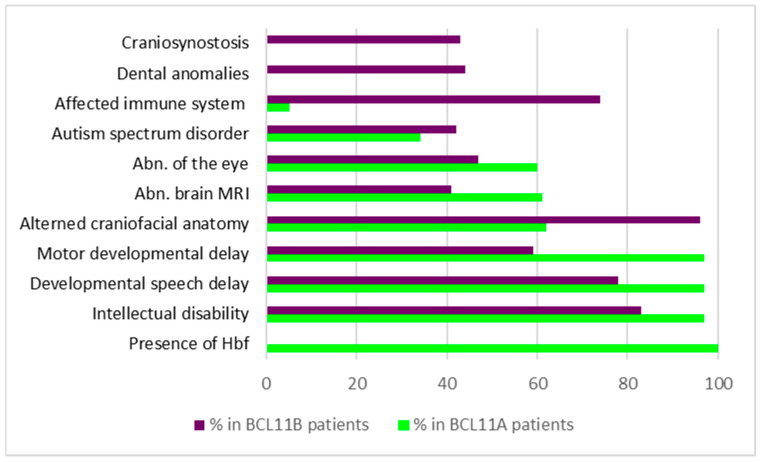
Symptoms and their prevalence in patients with variations in *BCL11A* or *BCL11B*, respectively. Data condensed from [13,48,62,63,64,65,66,67,68,69,70,71,72,73,74,75,76].

Symptoms occurring with the highest prevalence were elevated levels of fetal hemoglobin (100%), hypotonia (70%), strabismus (60%), and neurodevelopmental symptoms, including ID (97%), postnatal microcephaly (50%), delay in speech/gross/fine motor development (95%/96%/97%), and behavioral abnormalities (66%) (Figure 1) [51]. In addition, brain MRI analysis revealed structural changes in 61% of the individuals, including abnormalities of the posterior fossa (37%), the brainstem (31%), the corpus callosum (19%), and the cortex (4%). Other lower prevalence phenotypes like ASD (34%), seizures (24%), and musculoskeletal phenotypes, such as joint hypermobility (38%) and scoliosis (23%), were present in a subset of affected individuals. Moreover, mild craniofacial features, such as external ear abnormalities (62%), malar flattening (59%), and full cheeks (43%), were reported in several of the patients. Intriguingly, no prominent immune system dysfunction was observed in the entire cohort [51].

### 2.2. Functions of BCL11A in CNS Development

During the development of the CNS, Bcl11a is expressed in mice and humans within multiple regions known to be involved in NDDs, including the neocortex, hippocampus, and cerebellum [20,51]. Experiments in mice defined important functions of Bcl11a in multiple developmental processes, including neuronal migration, differentiation, and survival, as well as axonal growth and circuit formation [52,53,54]. Early studies showed that knockdown of Bcl11a in cultured neurons increased axon branching, multi-axon formation, and dendrite outgrowth, which could be rescued by the overexpression of deleted in colorectal carcinoma (Dcc) and microtubule-associated protein 1b (Map1b) [52]. Alternative splice forms of Bcl11a serve distinct functions in fine-tuning axonal branching and outgrowth in cell culture [29,52]. During spinal cord development, Bcl11a acts upstream of the secreted frizzled-related protein 3 (sFRP3, or Frzb) in neuronal morphogenesis and sensory wiring [53]. In the establishment of the neocortex, where the neuronal functions of Bcl11a were intensively studied, radial glia cells, intermediate progenitors, and young post-mitotic cells express Bcl11a from embryonic day (E) 12.5 onwards [54,55]. A recent study showed a decreased proliferation in radial glia cells upon conditional knock-out of Bcl11a and an accumulation of intermediate progenitor cells [55]. Bcl11a-deficient neurons fail to switch from multipolar to bipolar fate, resulting in impaired migration and a subsequent cortical layering phenotype [54]. In this context, Bcl11a represses its target semaphorin 3c (Sema3c) as a mediator of radial migration, and normalization of Sema3c in Bcl11a deficient neurons rescues the migration defects [54]. Later, during the postnatal development of the neocortex, the absence of Bcl11a in the cortex results in the downregulation of the antiapoptotic transcriptional repressor Bcl6, which promotes apoptosis of upper-layer neurons [56]. As a result of the reported incorrect cellular processes, the functional architecture and neuronal connectivity are impaired, which may be necessary for the acquisition of higher brain functions. Another interesting target gene of Bcl11a is the *t-box brain 1 TF* (*Tbr1*), which is highly expressed in the deep layers of the neocortex and was found to regulate the expression of cortical-related ASD genes, such as *Reln*, *Grin2b,* and *Aut2* [57,58]. Notably, patients carrying heterozygous variants of *TBR1* exhibit features of NDDs comparable to patients with BCL11A-IDD, such as speech and language impairment, ID, and ASD, as well as structural changes in the brain [59,60]. Consistent with this, Bcl11a and Tbr1 were identified in a transcriptional interactome (including, among others, Foxp2, Cask, and CoupTF1/2) that is important in neuronal development and associated with the occurrence of NDDs [60,61]. Besides its role in the neocortex, Bcl11a defines distinct subpopulations of dopaminergic neurons in the midbrain [62]. The deletion of Bcl11a in dopaminergic neurons increases their susceptibility to toxic insults and impairs motor development in mice [62].

**Figure 2:**
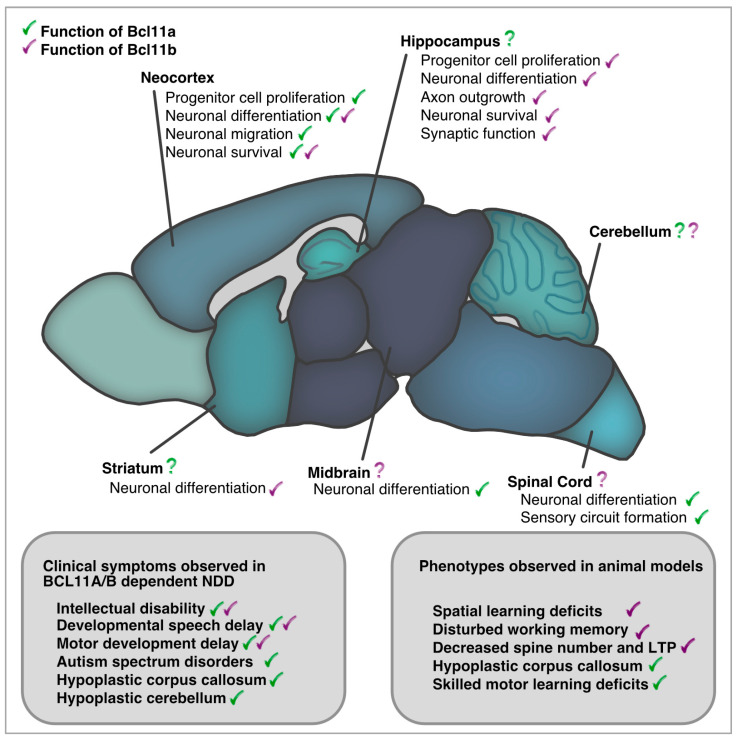
Bcl11a and Bcl11b in development and adult function of the central nervous system. Comparison of clinical phenotypes reported in BCL11A/B dependent NDDs and those described in animal models.

Taken together, in the nervous system—similar to the hematopoietic system—Bcl11a acts as a cellular coach, guiding progenitor and immature cells towards the next step of maturation and differentiation. Bcl11a controls transitions in transcriptional patterns that lead to morphological changes in cellular architecture, which may be important for the acquisition of higher brain functions. Although Bcl11a is expressed in several regions throughout the evolving CNS, particularly in regions known to be affected by NDDs, only a small number of these regions were thoroughly studied in detail for their functional roles (Figure 2).

### 2.3. BCL11B-Related NDDs

Based on the clinical manifestations described, the OMIM database lists variants in *BCL11B* as the cause of “Immunodeficiency 49” (OMIM: 617237) and “Intellectual developmental disorder with speech delay, dysmorphic facies, and T-cell abnormalities” (OMIM: 618092).

The initial case report documenting an individual with a *BCL11B* variation and BCL11-related disorder (BCL11-RD) was published by Punwani et al. in 2016. The patient presented at the hospital with a ‘leaky’ severe combined immunodeficiency (SCID; T-B + NK+). Following WES, a de novo missense mutation in *BCL11B* (p.Asn441Lys) was discovered. Medical examinations revealed a multitude of symptoms, such as erythematous psoriasiform dermatitis, neonatal teeth, craniofacial abnormalities, Wormian skull bones, hypotonia, and a significant developmental delay [44]. Beyond that, the brain MRI revealed callosal agenesis, hippocampal malformations, parallel configuration of the third ventricles, prominent occipital horns, and a general volume loss within the white matter.

Although hematopoietic stem-cell transplantation was successful in treating the SCID, the patient later developed ID with spastic quadriplegia and seizures, which are well-described features of NDDs. To date, 16 additional articles have been published, covering a total number of 57 individuals with variations in the *BCL11B* gene loci [14,63,64,65,66,67,68,69,70,71,72,73,74,75,76,77]. The reported genetic variants and clinical symptoms make a systematic genetic/phenotypic correlation of the *BCL11B* gene more feasible. In this cohort, 46 unique variants of *BCL11B*, including 25 frameshift, 12 missense, 1 splice-site, 4 stop-gain variants, and 6 unique translocations, were investigated. Except for two families in which genetic variants were maternally inherited, the majority occurred de novo.

Similar to the situation in BCL11A-IDD, ID (83%), delayed speech development (78%), and delayed motor development (58%) belonged to symptoms with the highest prevalence in patients with *BCL11B* variations (Table 3/Figure 1). In addition, patients frequently showed craniofacial dysmorphias (96%), including myopathic facial appearance, prominent nose, thin eyebrows, long philtrum, thin upper lip vermilion, small palpebral fissures, and hypertelorism. Although the immune system is affected in 74% of individuals carrying *BCL11B* variants, only a few develop severe immunodeficiency [14,44]. In contrast to LOF variants of *BCL11B*, patients with severe immunodeficiency typically harbor missense mutations in their genome, suggesting dominant-negative and/or gain-of-function mechanisms to be involved in the pathogenesis of disease [14]. Most of the existing truncation variants are thought to be degraded by the nonsense-mediated mRNA decay machinery, resulting in haploinsufficiency. Only truncation variants near the C-terminal end are predicted to escape degradation and result in shortened BCL11B proteins lacking C-terminal zinc finger domains.

A recent study determined two distinct DNA methylation profiles in the peripheral blood of BCL11B-IDD patients that show an overall hyper-methylation. These epigenetic signatures were defined to serve different aspects of diagnosis. The first was optimized for sensitivity and the second for specificity to enable clinicians to choose the appropriate profile according to underlying cases. These novel epigenetic signatures provide additional diagnosis tools and build a base for further elucidating the epigenetic impact of BCL11B in the future [73].

Besides its involvement in NDDs, BCL11B was previously linked to neurodegenerative diseases, such as Huntington’s disease, schizophrenia, Alzheimer’s disease, and amyotrophic lateral sclerosis (ALS), which indicates a role of BCL11B in the maintenance of functional neuronal connections [78,79,80,81].

### 2.4. Functions of Bcl11b in CNS Development

In the developing CNS, Bcl11b is expressed in the olfactory system, neocortex, hippocampus, entorhinal cortex, cerebellum, striatum, and spinal cord [20,82]. Here, Bcl11b is necessary for establishing and maintaining neuronal connections, which is associated with the etiology of NDDs [82,83,84,85]. Early investigations of homozygous Bcl11b knockout in mice, which do not survive beyond postnatal day 0, revealed major changes in axonal projections of cortico-spinal motor neurons [34,82]. Furthermore, cortico-spinal motor neurons show a reduced capacity for axon outgrowth and pruning in heterozygous Bcl11b mice [82]. In the striatum, where Bcl11b is expressed in postmitotic medium spiny projection neurons (MSN), Bcl11b regulates gene expression that is central to the differentiation of MSNs [86]. The loss of Bcl11b function leads to transcriptional changes of molecular identifier genes followed by a disturbed patch-matrix organization [86]. The function of Bcl11b within the formation of the hippocampus was comprehensively examined [83,87,88,89]. Bcl11b is expressed in the hippocampus throughout development and in adulthood. Initially, Bcl11b is expressed at E15 in the progenitor cells of the cornu ammonis (CA). Furthermore, it is present in postmitotic dentate granule cells and pyramidal cells of CA1 and CA2 during development and adulthood. Its presence is important for cell proliferation, differentiation, and maintenance within the DG, as well as the functional integration of dentate granule cells [83,87]. Conditional forebrain-specific Bcl11b knockout mice show a hypoplastic DG, reduced number of dendritic spines, disturbed mossy fiber tract, and behavioral abnormalities, including impaired spatial learning and memory [83]. Similar phenotypes were also described in mice with the conditional knockout of the cell adhesion molecule desmoplakin, the expression of which was found to be directly dependent on Bcl11b [83]. Furthermore, the selective removal of Bcl11b in the adult forebrain results in a loss of synapse numbers and long-term potentiation at mossy fiber synapses [88]. In addition, the ultrastructural complexity of the synapses is lost, and the synaptic vesicles are disorganized upon the loss of Bcl11b [88]. In line with this, a recent study identified the synaptic organizer molecule C1ql2 as a direct functional target of Bcl11b at the mossy fiber synapses [89]. Interestingly, C1ql2 regulates synaptic vesicle recruitment to the active zone through direct interaction with Nrxn3(25b+), a specific splice variant of neurexin-3 that was linked to NDDs before [89,90]. Changes in synaptic signal transduction, including establishment, transport, and release of synaptic vesicles, as well as alterations on the postsynaptic side are known to be hallmarks of NDDs [91,92,93]. Besides its role in development and maintenance of neocortex and hippocampus, Bcl11b is present in postmitotic but immature neurons of the vomeronasal sensory system. Here, Bcl11b is also important for differentiation and axon guidance, and upon the ablation of Bcl11b, vomeronasal sensory neurons fail to build their axonal projections and undergo cell death [84].

Taken together, both Bcl11 TFs have principal functions during nervous system development. While Bcl11a mediates predominantly transitions in gene expression patterns to switch neuronal identity, Bcl11b mediates differentiation of neurons but, beyond that, controls synaptic vesicle organization and, thus, has a direct impact on intercellular communication (Figure 2). The loss of Bcl11b leads to alterations in axons and synapses, resulting in dysfunctional signaling—a process critical for advanced brain function and a major determinant of NDDs.

### 2.5. Bcl11a and Bcl11b in CNS Development

Even though Bcl11a and Bcl11b have different exon-intron structures, they share six highly related zinc-finger domains that suggest a commonality of function [16]. In evolutionary terms, Bcl11b is segregated from a Bcl11a homolog during the occurrence of vertebrates [19,94]. In *Drosophila melanogaster*, for example, only one homolog of Bcl11a and Bcl11b exists, evolutionary closer to Bcl11a, which is called Chronophage (Chp). Chp is expressed in some neurons of the ventral nerve cord and larval CNS and is involved in temporal patterning and neuronal subtype specification during early embryogenesis [18]. Here, Cph promotes the expression of Castor, which belongs to a cascade of temporally successive TFs with unique temporal windows [95]. In *Cph* null mutants, neuronal precursors fail to undergo the (early) Pdm to (late) Castor transition, and larvae die in the late stages of embryogenesis [18]. Fruit flies carrying a neuron-specific knockdown of *Cph* are viable and fertile but show structural changes in larval synapse organization, such as an increased number of synaptic branches in the neuromuscular junction. In addition, Yamaguchi et al. show NDD-like phenotypes in these flies. Larvae display locomotor defects and reduced learning ability, whereas epileptic-like behavior was observed in adult flies [96].

Although NDD patients only have heterozygous mutations in either *BCL11A* or *BCL11B*, it is of mechanistic interest to observe the double mutant situation. Recently, the joint action and crosstalk of Bcl11a and Bcl11b in neocortical development was examined [55]. In the neocortex, Bcl11a is important for the migration of neural progenitors, and Bcl11b is present in a subset of glutamatergic cortical neurons as well as interneurons [54,82,97]. The knockout of both Bcl11a and Bcl11b results in an exacerbated phenotype, including defective cortical axon tracts, massive cell death of cortical neurons, and a severe hypoplastic cortex [55]. Bcl11a additionally regulates the specification of neuronal subtypes in projection neurons, while Bcl11a and Bcl11b redundantly promote the identification of neuronal subtypes [55,98].

The reciprocal functions of Bcl11a and Bcl11b in other brain regions where they are (transiently) co-expressed have not yet been examined and represent a fertile area for future studies. In the hippocampus, for example, Bcl11b is an important regulator of neurogenesis and neuronal differentiation during development [83]. While Bcl11a is expressed during hippocampal development, its functions both autonomously and in crosstalk with Bcl11b are unclear [20]. Also, in the cerebellum, where Bcl11a and Bcl11b are present in progenitor and adult Purkinje cells, redundant functions may be possible and remain to be identified [20,51].

## 3. BCL11 TFs in Animal Models of NDDs

NDDs are caused by disturbed brain development that results in impaired motor, cognitive, social, and language function [99]. Pathomechanisms of brain development underlying NDDs are fairly understood and investigated so far. Three factors are of special relevance for the modeling of genetic human disorders in animals: (i) strong genetic conservation, (ii) high face validity, and (iii) robustness and reproducibility of phenotypes [100]. At a minimum, these three conditions must be met in models to ensure reliable translation from patient to model and vice versa. In the case of BCL11 TFs, all factors are approved to some extent. The first factor since the loci of Bcl11 TFs resemble ultra-conserved gene regions [15,17]. The second factor of face validity is demonstrated by comparable structural, physiological, and behavioral phenotypes observed in both, patient and model systems. The third factor is critical in identifying relevant experiments for the evaluation of prospective clinical targets. Several approaches were taken in which animal models with altered or missing Bcl11 genes serve as in vivo models for NDDs (Table 4) [13,14,44,75,96]. NDD-like phenotypes could be observed in *Drosophila melanogaster* throughout evolutionarily advanced organisms [13,83,87,96]. There are genetic mouse models that reproduce the anatomical phenotypes of NDD patients, such as microcephaly and smaller skulls [13]. Furthermore, these mouse models can reflect the neurodevelopmental symptoms of BCL11 loss in behavior studies. Mice show, among other things, long-term social memory deficits, impaired sociability, and increased activity [13,83,87]. The extent and origin of structural and functional changes in mice can be studied using biochemical, molecular, and histological methods [13,83]. Another article in this issue describes the generation of mouse alleles in which fluorescent reporter and affinity tags were added to *Bcl11a* and *Bcl11b*, respectively [101]. The novel mouse lines will simplify visualization and cell sorting of Bcl11 TF expressing cells and can be used in immunoprecipitation and pull-down assays, therefore providing promising tools to further analyze the molecular and biochemical role of Bcl11 TFs in future studies [101]. In addition to classical genetic animal models, humanized animal models are becoming more common for unraveling disease processes and will be helpful in identifying suitable drug targets in the future. Human mutant variants of *BCL11A* and *BCL11B* were used to investigate phenotypes and perform rescue experiments in zebrafish [44,75]. Genome-editing techniques utilizing CRISPR-Cas9, including *i*-GONAD (improved genome editing via oviductal nucleic acid delivery), are useful for inserting patient-derived variants into genes of interest [102]. For example, Goos et al. applied CRISPR-Cas9 to introduce a patient variant of *BCL11B* (p.Arg3Ser BCL11B) into the germline of C57BL/6 mice. The patient did not exhibit obvious signs of NDDs during early childhood but displayed craniofacial abnormalities, craniosynostosis, vision impairment, and an epileptic seizure occurring when he was 11 years old [63]. The humanized heterozygous BCL11B^p.Arg3Ser/+^ mouse model, in part, phenocopied the human situation by a non-closure of cranial sutures, which was also observed in Bcl11 knockout mice [63,103].

In summary, the research conducted on Bcl11 TFs demonstrates that all three modeling factors are fulfilled and that animals provide powerful models for investigating the molecular functions of Bcl11 TFs in NDDs. However, there are still regions of the brain that are known to be affected by NDDs but were not functionally studied in relation to Bcl11a or Bcl11b. One example is the cerebellum, which is known to be important for fine- and gross-motor coordination but also serves cognitive functions and affective regulation and is, therefore, often affected in NDDs [104,105]. BCL11 TFs are expressed during cerebellar development by distinct neuron populations. Fine- and gross-motor development is delayed in most patients with variations in both *BCL11A* or *BCL11B* genes [14,20,50,51]. Moreover, marked cerebellar hypoplasia was reported in patients with *BCL11A* mutations [51]. Yet, the cellular and molecular functions of Bcl11a in cerebellar development were not analyzed experimentally. However, it is tempting to speculate that the clinical phenotype results from a dysregulation of developmental mechanisms, including the proliferation, migration, or differentiation of cerebellar neurons, which may, in turn, lead to impaired integration into functional neuronal networks. As the development of the cerebellum is largely postnatal, the system holds great promise for future therapies. Hence, systematic studies on transcriptional regulation, neuronal and structural alterations, as well as behavioral phenotyping need to be established. For example, a standardized set of behavioral tests was recently evaluated for further application in the preclinical testing of NDDs [106]. The set comprises the open field test, marble burying test, nest building test, rotarod, and forced swim test and can be adapted for modeling variations in *BCL11A* or *BCL11B*, respectively. One more research target involves the implementation of two-hit models since the pre- and postnatal environment is known to affect brain development [3,107]. Maternal immune activation, stress, undernutrition, drug exposure, and delivery complications, as well as later febrile convulsions, can all interfere with the development of structures and circuits in the CNS [3]. However, the additive effects of genetic and environmental influences are still not adequately understood.

## 4. Conclusions—Future Directions

Neural development requires the precise and delicate orchestration of diverse regulatory cascades in a spatiotemporal-dependent manner to achieve a highly ordered functional network. The dysregulation of processes, such as transcription and translation during neurodevelopment, typically impairs the neurogenesis, migration, or differentiation of neurons, as well as the wiring and function of synapses [4]. TFs serve as key molecular controls over these processes by regulating gene availability and expression in chromatin remodeling complexes. Emerging evidence sheds light on the two members of the BCL11 TF family, namely Bcl11a and Bcl11b, in this regard. In mice, both TFs were reported to epigenetically modify processes crucial for neurodevelopment, such as proliferation, maturation, and survival [54,83]. As a consequence, the deletion of Bcl11a or Bcl11b leads to cellular, neurostructural, and behavioral alterations in mice [13,83]. Over the past decade, a growing number of patients have been identified with NDDs that carry mutations in *BCL11A* or *BCL11B*. In most cases, the patients exhibit a high prevalence of ID, motor, as well as speech developmental delay and display neurostructural changes [14,51,73]. Those striking similarities of developmental malformations observed in both mice and humans favor the implementation of modeling human diseases in animals to elucidate mechanistic functions of Bcl11 TFs in the future [13,63,108]. Furthermore, genetic studies in mice were shown to provide a promising approach for better understanding the roles of Bcl11a and Bcl11b for physiological as well as pathophysiological mechanisms underlying NDDs. The modeling of the disorder in mice and simpler models, such as zebrafish and fruit flies, was already performed and provided powerful tools for future research. High throughput analysis for examining molecular mechanisms necessitates the use of relatively simple and cost-effective models. Candidate targets and their impact on morphogenesis can be further investigated using more complex models, including conditional knockout mice or humanized mice. With them, the systematic investigations of phenotypes previously documented in patients, such as developmental delays in cognitive, language, and motor development, and their related structural modifications, such as those in the cortex, the hippocampus, and the cerebellum, must be performed. Moreover, extensions of WES and epigenetic signatures in routine clinical practice will contribute to a broader recourse of individuals with variants in *BCL11A* or *BCL11B*. This offers a valuable opportunity to further decipher genotype/phenotype correlations and the potential pathological mechanisms that drive clinical phenotypes. By conducting more comprehensive and systematic investigations of previously identified patients, we can strengthen diagnostic and counseling strategies, improve prognoses, and even develop more causative therapies for certain symptoms. To ensure a proper translation of these therapies into clinical trials, mouse models that exhibit high similarity at the genotypic and phenotypic levels must be conscientiously established and investigated for future applications.

## Figures and Tables

**Table 1:** Neurodevelopmental disorders as described in the International Statistical Classification of Diseases and Related Health Problems (ICD-11).

ICD-11 Neurodevelopmental Disorders
**6A00 Disorders of intellectual development**
6A00.0 mild; 6A00.1 moderate; 6A00.2 severe; 6A00.3 profound; 6A00.4 provisional
**6A01 Developmental speech or language disorders**
6A01.0 Developmental speech sound disorder; 6A01.1 Developmental speech
fluency disorder
**6A02 Autism spectrum disorder (ASD)**
6A02.0 without disorder of intellectual development and with mild/no impairment
of functional language; 6A02.1 with disorder of intellectual development and with
mild/no impairment of functional language; 6A02.2 without disorder of intellectual
development and with impairment of functional language; 6A02.3 with disorder of
intellectual development and with impairment of functional language; 6A02.5 with
disorder of intellectual development and with absence of functional language
**6A03 Developmental learning disorder**
6A03.0 with impairment in reading; 6A03.1 with impairment in written expression;
6A03.2 with impairment in mathematics; 6A03.3 with other specified impairment in
learning
**6A04 Developmental motor coordination disorder**
**6A05 Attention deficit hyperactivity disorder (ADHD)**
6A05.0 predominantly inattentive presentation; 6A05.1 predominantly hyperactive-
impulsive presentation; 6A05.2 combined presentation
**6A06 Stereotyped movement disorder**
6A06.0 without self-injury; 6A06.1 with self-injury

**Table 2:** Profile of transcription factors Bcl11a and Bcl11b.

	Bcl11a	Bcl11b
**Gene ID**	53335	64919
**NCBI RefSeq**	NM_022893.4	NM_138576.4
**Synonyms**	Ctip1, Dilos, Evi9, Hbtqtl5, Smarcm1, Znf856	Atl1, Ctip2, Iddfsta, Imad49, Rit1, Smarcm2, Znf856b
**Human Isoforms**	XL (835 aa) L (773 aa) S (243 aa)	Alpha (823 aa) Beta (812 aa)
**Isoforms in mice**	XL (835 aa) L (773 aa) S (243 aa) XS (191 aa)	Alpha (884 aa) Beta (882 aa) Gamma (690 aa)
**#MIM**	606557	606558
**#Phenotype (MIM)**	617101 (Dias-Logan syndrome) 612513 (Chromosome 2p16.1-p15 deletion syndrome)	617237 (Immunodeficiency 49, severe combined) 618092 (Intellectual developmental disorder with dysmorphic facies, speech delay, and T-cell abnormalities
**#sysndd**	1235	2475, 2476

**Table 3:** Symptoms and their prevalence in patients with variations in *BCL11B*. Data condensed from [13,62,63,64,65,66,67,68,69,70,71,72,73,74,75,76].

Symptom	+	−	+/n	%	n.a.
Intellectual delay (ID)	45	9	45/54	83.3	3
Developmental speech delay	40	11	40/51	78.4	6
Motor development delay	30	22	30/52	57.7	5
Altered craniofacial anatomy	43	2	43/45	95.6	12
Abn. brain MRI	14	20	14/34	41.2	23
Abn. of the eyes	18	24	18/42	42.9	15
Autism spectrum disorders (ASD)	11	15	11/26	42.3	31
Affected immune system	34	12	34/46	73.9	11
Dental anomalies	14	18	14/32	43.8	25
Craniosynostosis	13	17	13/30	43.3	27

**Table 4:** Nonprimate animal models for investigation of Bcl11 TF-related NDDs.

Genetic Model	Species	References
*Bcl11a* ^+/−^	*Mus musculus*	[77]
*Bcl11a ^flox/flox^DatIRES-Cre/+*	*Mus musculus*	[62]
*Bcl11ab* knockdown *BCL11A p.Cys826Tyr*	*Danio rerio*	[75]
Neuron-specific *Chronophage* knockdown	*Drosophila melanogaster*	[96]
*Bcl11b^flox/flox^Emx1^Cre^* *Bcl11b^flox/flox^CamkTetOCre*	*Mus musculus*	[83,87,88,89]
*BCL11B p.Asn441Lys*	*Danio rerio*	[44]
BCL11B p.Gly820Alafs*67	*Mus musculus*	[14]
*BCL11B p.Arg3Ser*	*Mus musculus*	[63]

## Data Availability

Data contained within the article.
